# A Plant Endophytic Bacterium *Priestia megaterium* StrainBP-R2 Isolated from the Halophyte *Bolboschoenus planiculmis* Enhances Plant Growth under Salt and Drought Stresses

**DOI:** 10.3390/microorganisms10102047

**Published:** 2022-10-17

**Authors:** Hau-Hsuan Hwang, Pei-Ru Chien, Fan-Chen Huang, Pin-Hsien Yeh, Shih-Hsun Walter Hung, Wen-Ling Deng, Chieh-Chen Huang

**Affiliations:** 1 Department of Life Sciences, National Chung Hsing University, Taichung 402, Taiwan; 2 Innovation and Development Center of Sustainable Agriculture, National Chung Hsing University, Taichung 402, Taiwan; 3 Institute of Plant and Microbial Biology, Academia Sinica, Taipei 115, Taiwan; 4 Department of Plant Pathology, National Chung Hsing University, Taichung 402, Taiwan

**Keywords:** plant endophytic bacteria, drought stress, salt stress, *Priestia megaterium*, *Bacillus megaterium*

## Abstract

Global warming and climate change have contributed to the rise of weather extremes. Severe drought and soil salinization increase because of rising temperatures. Economically important crop production and plant growth and development are hindered when facing various abiotic stresses. Plant endophytic bacteria live inside host plants without causing visible harm and can be isolated from surface-sterilized plant tissues. Using plant endophytic bacteria to stimulate plant growth and increase environmental stress tolerance has become an alternative approach besides using the traditional breeding and genetically modifying approaches to select or create new crop types resistant to different environmental stresses. The plant endophytic bacterium, *Priestia megaterium* (previously known as *Bacillus megaterium*) strain BP-R2, was isolated from the surface-sterilized root tissues of the salt marsh halophyte *Bolboschoenus planiculmis*. The bacteria strain BP-R2 showed high tolerance to different sodium chloride (NaCl) concentrations and produced the auxin plant hormone, indole acetic acid (IAA), under various tested growth conditions. Inoculation of *Arabidopsis* and pak choi (*Brassica rapa* L. R. Chinensis Group) plants with the strain BP-R2 greatly enhanced different growth parameters of the host plants under normal and salt and drought stress conditions compared to that of the mock-inoculated plants. Furthermore, the hydrogen peroxide (H_2_O_2_) content, electrolyte leakage (EL), and malondialdehyde (MDA) concentration accumulated less in the BP-R2-inoculated plants than in the mock-inoculated control plants under salt and drought stresses. In summary, the plant endophytic bacterium strain BP-R2 increased host plant growth and stress tolerance to salt and drought conditions.

## 1. Introduction

Unlike animals, plants are stationary organisms that cannot change their growth environments and they constantly encounter different biotic and abiotic stresses, including salinity, drought, and extreme temperatures. Soil salinity and water deficiency are two major limiting factors for crop production worldwide that may continuously intensify due to climate change and global warming. Most global fresh water is currently used for agricultural practice and crop production. Extensive and improper irrigation practices also lead to salt accumulation in the soil. It is estimated that around 20 to 50% of irrigated lands are seriously affected by salinity [1,2,3]. Furthermore, salinity in agricultural lands is caused by insufficient rainfall and high water evaporation rates that result from rising temperatures and climate changes [2,3]. Both drought and salt stresses are multidimensional stresses that have profoundly negative effects on morphological, physiological, biochemical, and molecular processes in plants [4,5,6]. Drought stress may lead to a reduction in the water content of plant cells, cell division, cell growth, photosynthesis, respiration, plant hormone production, and sugar and nutrient metabolism [2]. Similarly, the accumulation of salt in soil may decrease the soil water potential, thereby negatively affecting water and ion absorption by plant roots, and finally leading to decreases in ion homeostasis, enzyme activities, nutrient uptake, plant growth, and crop production [5,6]. Thus, improving the drought and salt tolerance of plants is of great importance to the sustainable development of agriculture. Traditional plant breeding and genetic transformation approaches to create salt or drought tolerant crop varieties are cost-intensive and sometimes may be insufficient to alleviate abiotic stresses [7]. The application of plant growth-promoting microbes, such as beneficial rhizosphere, rhizoplane, phyllosphere, and endophyte bacteria, to enhance salt or drought tolerance in plants, may provide an alternative approach [7,8,9,10,11].

Plant endophytic bacteria live within host plants during different stages of their life cycles without causing infection and harmful effects to the host plants [12,13]. These bacteria are frequently isolated from surface-sterilized plant tissues or from internal plant tissues and usually enter plants from root wounds and cracks after a rhizospheric population is first established in the soil [12,14]. They can remain at the entry points, though a few can occupy the aerial parts of plants, such as leaves, flowers, and fruits. In host plants, higher numbers of endophytic bacteria exist in roots compared to that in stems and leaves [15,16]. The compositions of the endophytic bacteria populations differ, which depend on various factors including host plant ages, genotypes, and environmental growth conditions, analyzed plant tissue types, microorganism species, soil textures, pH, and contents, and other environmental factors [13,17]. The molecular identification of endophytic bacteria is frequently performed with sequencing of the 16S ribosomal RNA (16S rRNA) gene or with marker analysis methods, such as restriction fragment length polymorphism (RFLP) and denaturing gradient gel electrophoresis (DGGE) [12,13]. So far, more than 200 genera of bacteria have been identified as endophytic bacteria, and most of them belong to the Proteobacteria, Firmicutes, Actinobacteria, and Bacteroidetes phyla. The frequently isolated bacteria genera are *Azoarcus*, *Bacillus*, *Burkholderia*, *Enterobacter*, *Herbaspirillum*, *Microbacterium*, *Micrococcus*, *Pantoea*, *Pseudomonas*, *Serratia*, *Streptomyces*, and *Stenotrophomonas*, and the two predominant genera consist of *Bacillus* and *Pseudomonas* [12,14]. Some endophytes seem to be latent pathogens, and infections may occur under certain circumstances, such as other microorganisms interacting with the endophytes and changes in environmental conditions [14,16,17].

After successful plant colonization, the beneficial endophytic bacteria facilitate plant growth and provide protection through various direct and indirect mechanisms, which include atmospheric nitrogen fixation, macronutrient (such as phosphorous, potassium, iron, and zinc) solubilization; plant hormone, siderophore, hydrogen cyanide, and ammonia synthesis; and plant hormone level and response modulation as direct mechanisms [12,13,14]. The indirect mechanisms involve the production of various antibiotics, toxins, lytic enzymes, exopolysaccharide (EPS), biofilms, antimicrobial volatile organic compounds; and the formation of induced systemic resistance (ISR) responses in plants to inhibit infection and promote plant growth [18,19,20]. When plants encounter drought or salt stresses, plant endophytes can regulate the accumulation of osmolytes (compatible solutes), such as proline and sugars, and induce the expression of stress-associated genes and enzymes, such as superoxide dismutase (SOD) and catalase (CAT), to reduce oxidative damage and enable plants to withstand abiotic stresses [3,10,13]. Additionally, endophytic bacteria can produce auxin to increase root length, surface area and growth, and the numbers and lengths of lateral roots and may improve the tolerance of host plants to various abiotic stresses [3,8,10].

When plants grow in severe salinity and drought environments, the presence of endophytic bacteria in these plants may help them to survive and grow in these conditions. Several studies have shown the beneficial effects of endophytic bacteria on their host plants, such as the *Salicornia brachiate* [21] plant, *Cicer arietinum* [22], *Haloxylon ammodendron* [23], *Nypa fruticans* [24], *Salicornia bigelovii* [25], *Armoracia rusticana* [26], *Seidlitzia rosmarinus* [27], *Calendula officinalis* [28], *Halocnemum strobilaceum* [29], *Haloxylon aphyllum* [30], *Tetragonia tetragonioides* [31], and other plants. In this study, we have isolated an endophytic bacterium from the salt marsh halophyte, *Bolboschoenus planiculmis*. The *Bolboschoenus planiculmis* plant is a wetland sedge that has great ecological and agricultural values. *B. planiculmis* plants are distributed towards the westernmost regions of Spain, easternmost areas in Japan, southernmost areas towards New Guinea, and northernmost regions towards Russia [32]. Plants of the genus *Bolboschoenus* are distributed in a wide range of habitats, including coastal and inland salt marshes, littoral zones of freshwater bodies, and temporarily flooded arable land [33,34]. The *B. planiculmis* species is a perennial plant that has a highly branched underground rhizome with spherical or elongated tubers that confer a competitive ability to reproduce quickly over other plants [35]. Although the salt tolerance of *B. planiculmis* had been tested in previous ecological studies [33,34,36], its mechanism of adapting to the high salinity remains largely unknown. Unlike the stratified distribution caused by interspecies competition in Europe, the *B. planiculmis* plants develop a circular colony that scatters through the coastal salt marsh in Taiwan. The *B. planiculmis* plant has become an endangered species in Taiwan due to over exploitation of the coastal land. With its high fitness to abiotic stress, such as drought and salinity [37], the *B. planiculmis* species may serve as a good candidate host plant to identify beneficial plant endophytes.

In order to identify beneficial bacteria that may promote plant growth and increase tolerance to salt stress, we have used the salt marsh halophyte, *B. planiculmis*, as a host plant and successfully identified a beneficial plant endophytic bacterium, strain BP-R2, in this study. We have observed its ability to tolerate different concentrations of sodium chloride (NaCl). Relatively high amounts of the auxin plant hormone, indole acetic acid (IAA), were produced by the strain BP-R2 under various growth conditions. Furthermore, the inoculation of *Arabidopsis* or pak choi (*Brassica rapa* L. R. Chinensis Group) plants with the strain BP-R2 significantly improved host plant growth under salt and drought stress conditions. This study effectively demonstrated that the plant endophytic bacterium, strain BP-R2, has the potential to be used as a bioinoculant in agricultural practices to enhance plant growth and salt and drought tolerance. In addition, this was the first study to show that a beneficial plant endophytic bacterium can be isolated from the *B. planiculmis* plant, the endangered plant indigenous to Taiwan and its possible ecological functions of this strain to help host plants survive in the harsh living environment.

## 2. Materials and Methods

### 2.1. Isolation and Identification of the Endophytic Bacteria from the Bolboschoenus planiculmis Plants

The endophytic bacteria were isolated and characterized according to the protocols of Ho et al. (2015) [38] and Hwang et al. (2021) [39]. The *Bolboschoenus planiculmis* plant samples were collected from the estuary soil of Dajia River at Taichung, Taiwan, located in a subtropical climate region with around 24 °C in annual average temperature. The soil pH values of the collected site ranged from 6.2 to 8.3, and the electrical conductivity of the soil solutions were about 0.6 to 12.0 mmhos/cm. The surface-sterilized root tissues were ground in sterilized mortars and the homogenates were serially diluted to spread on the Luria Broth (LB) agar medium (1.0% tryptone, 0.5% yeast extract, 0.5% NaCl, pH 7.5) at 30 °C. The bacteria identity was determined by sequencing and phylogenetic analysis of the 16S rRNA gene. The 16S rRNA gene amplification was obtained with colony PCR reactions using primers E8F (5′-AGAGTTTGATCATGGCTCAG-3′) and U1510R (5′-CGGTTACCTTGTTACGACTT-3′) [39]. The 16S rRNA sequences were analyzed by the basic local alignment search tool (BLAST) program compared with the GenBank database from the National Center for Biotechnology Information (NCBI) to identify the closest matching sequence. The partial 16S rRNA gene sequences of the isolated endophytic bacteria from the *Bolboschoenus planiculmis* plant were deposited in the GenBank database under accession number OP209759.

### 2.2. Physiological and Biochemical Characterization of the Endophytic Bacteria Strain BP-R2

The endophytic bacteria strain BP-R2 was isolated from the roots of the *Bolboschoenus planiculmis* plant. For growth tests of strain BP-R2 in various conditions, bacteria were grown in LB media at 20 °C, 25 °C, 30 °C, 37 °C, or 45 °C; or in LB media with a pH of 4.0, 5.7, 6.0, 7.5, or 9.0; or in LB media with 0.5%, 1.0%, 2.0%, or 3.0% sodium chloride (NaCl); or in *Agrobacterium* minimal essential (AB-MES) media [40] with 2% glucose, fructose, sucrose, galactose, lactose, raffinose, mannose, or starch; or in AB-MES media with 2% glucose and 0.5% beef extract, casein, tryptone, peptone, urea, ammonium sulphate, ammonium nitrate, or ammonium chloride at 30 °C. Bacterial cells were measured at OD_600_ to determine the biomass and were then grown for 48 or 72 h. Three biological replicates were used for each growth measurement.

The fatty acid composition of strain BP-R2 was determined using the MIDI Sherlock Microbial Identification System (Microbial Identification Inc., Newark, DE, USA) according to the manufacturer’s instructions and the protocol of Hwang et al. (2013) [41]. The bacterial strain was grown on trypticase soy broth (TSB) agar (3% TSB, 1.5% agar) at 28 °C for 2 days and was used for sample extraction. Fatty acid methyl esters (FAMEs) were extracted from bacteria and were analyzed by gas chromatography-mass spectrophotometry. The MIDI Sherlock MIS v6.0 software (Microbial Identification Inc., Newark, DE, USA) was used to compare the FAME profile of the bacteria to data in a stored database for bacteria identification.

The Biolog tests (Biolog Inc., Hayward, CA, USA) were used to determine the utilization patterns of 95 carbon substrates of the isolated bacteria according to the manufacturer’s instructions and the protocol of Hwang et al. (2013) [41]. Bacterial strains were grown on Biolog Universal Growth (BUG) agar medium (5.7% BUG agar) at 30 °C for 2 days, and were collected, resuspended, and adjusted to a suitable turbidity in Gram-negative/Gram-positive inoculating fluids (0.01% gellan gum, 0.4% NaCl, and 0.3% Pluronic F-68) according to the manufacturer’s instructions. The bacterial suspension solutions were applied to the GN2 microplates and were incubated at 28 °C for 24 to 48 h. The Biolog Microstation reader was used to record the positive color reactions, and 95 carbon substrates utilization patterns were analyzed and compared with the database of Biolog GN v4.0 (Biolog Inc., Hayward, CA, USA) for bacterial identification.

### 2.3. Analysis of Indole Acetic Acid (IAA) Production by the Colorimetric Assay

IAA production was analyzed by the Salkowski method as described by Hwang et al. (2021) [39] with minor modifications. The bacteria strain BP-R2 was grown in LB medium containing 100 μg/mL of tryptophan at different temperatures (20 °C, 25 °C, 30 °C, 37 °C, or 45 °C); or at a different pH (pH 4.0, 6.0, 7.5, or 9.0) at 30 °C; or with different NaCl concentrations (0.5%, 1.0%, 2.0%, or 3.0% NaCl) at 30 °C for 48 h; or in AB-MES media with 100 μg/mL of tryptophan, 2% of various carbon sources, and 0.5% of different nitrogen sources with a pH of 7.5 at 30 °C for 72 h. The supernatants of the bacteria cultures were collected, filtered to remove the bacteria, and mixed with the Salkowski reagent. Finally, the IAA concentrations were determined by an absorbance measured at 530 nm (OD_530_) and compared with a standard curve of 0–100 µg/mL IAA.

### 2.4. Inoculation of Plants with the Endophytic Bacteria Strain BP-R2 and Measurement of Plant Growth Parameters

The bacteria inoculation and re-isolation assays were performed as described by Hwang et al. (2021) [39] with minor modifications. The four- to six-leaf seedlings of wild-type *Arabidopsis thaliana* (ecotype Columbia) and pak choi (*Brassica rapa* L. R. Chinensis Group) grown in pots were inoculated with bacteria strain BP-R2. The bacteria cultures grown in LB media at 30 °C to an approximate OD_600_ value of 0.6–0.8 were used for the inoculations. After inoculation, plant seedlings were continually grown in soil at green houses for further salt and drought stress treatments. For salt stress treatments, the BP-R2-inoculated *Arabidopsis* and pak choi plants were treated with 250 mM NaCl and 200 mM NaCl for 5 days, respectively. The negative control plants were treated with distilled water instead of NaCl. Subsequently, the NaCl-treated plants were treated with distilled water and recovered for 3 days. For drought stress treatments, both BP-R2-inoculated *Arabidopsis* and pak choi plants were treated with dehydration for 10 and 5 days by withholding water, respectively. The plants were subsequently re-watered for 3 days for recovery. After salt and drought stress treatments, several plant growth parameters, including the fresh weight (FW), dry weight, rosette diameter, number of leaves, length of leaves, and surface area of leaves, were measured in harvested *Arabidopsis* and pak choi plants as described previously [39]. The *Arabidopsis* or pak choi plants that were not inoculated with strain BP-R2 and not treated with salt or drought stress were used as the negative controls.

To verify the endophytic colonization of the inoculated plants with strain BP-R2, surface-sterilized plant tissues were ground with sterile distilled water and plant crude extracts were serially diluted and plated on LB agar to determine the viable bacteria cell numbers. The sequencing and phylogenetic analysis of the 16S rRNA gene was conducted to determine the bacteria identity. Colony PCR reactions were performed with primers E8F (5′-AGAGTTTGATCATGGCTCAG-3′) and U1510R (5′-CGGTTACCTTGTTACGACTT-3′) of the 16S rRNA gene [39].

### 2.5. Hydrogen Peroxide (H_2_O_2_) and Proline Content Determination

Leaves from the mock-inoculated control and the bacteria-inoculated, stress-treated plants were collected and ground to determine the hydrogen peroxide (H_2_O_2_) concentrations by a colorimetric reaction with xylenol orange (XO) according to Huang and Hwang (2020) [42]. H_2_O_2_ concentrations were determined by an absorbance measured at 560 nm (A_560_) by comparison with a standard curve of 0–100 µM H_2_O_2_.

The proline content was determined using the method of Bates et al. (1973) [43] with minor modifications. Leaves were first homogenized with 3% sulfosalicylic acid, and the supernatant was mixed with the acidic ninhydrin reagent and heated to 100 °C. Finally, the reaction mixture was extracted with toluene after cooling. The proline concentration was then determined by an absorbance measured at 520 nm (A_520_) by comparison with a standard curve of proline.

### 2.6. Electrolyte Leakage (EL) and Malondialdehyde (MDA) Content Determination

The EL amounts of the leaf samples were measured according to Gontia-Mishra et al. (2016) [44] with minor modifications. Fresh leaves from the mock-inoculated control and the bacteria-inoculated, stress-treated plants were kept in closed tubes containing suitable amounts of deionized water. The electrical conductance (EC1) of the solution was measured after the plant samples were placed on a rotary shaker at room temperature for 24 h. Leaf samples were then autoclaved at 121 °C for 20 min and the final electrical conductance (EC2) was determined. The electrical conductance (ECnc) of the deionized water without plant tissues was used as a negative control. The electrolyte leakage (EL) was calculated using the following equation: EL (%) = (EC1-EC1nc/EC2-EC2nc) × 100.

The MDA concentrations of the leaf samples were measured according to Hodges et al. (1999) [45] with minor modifications. Leaves were first ground and homogenized in ice-cold 80% ethanol. The plant crude extract was then mixed with 20% (*w*/*v*) trichloroacetic acid (TCA) containing 0.5% (*w/v*) thiobarbituric acid (TBA) and was subsequently heated at 95 °C for 30 min, quickly cooled, and finally centrifuged at 10,000× *g* for 10 min. The MDA concentration was determined by absorbances measured at 440 nm, 532 nm, and 600 nm. The MDA content was measured as μmol/g fresh weight.

### 2.7. Statistical Analysis

The plant growth measurements were average values from at least three independent bacteria inoculation experiments. At least 20 different seedlings of *Arabidopsis* or pak choi plants were inoculated with strain BP-R2, and more than 60 individual plants were examined for bacteria inoculation assays. Error bars were calculated with the Microsoft Excel (Microsoft Corporation, Redmond, WA, USA) STDEVP function. The significance test between treatments was based on Duncan tests, with *p* < 0.05 considered statistically significant.

## 3. Results

### 3.1. Isolation and Characterization of the Endophytic Bacteria Strain BP-R2 from the Roots of Bolboschoenus planiculmis Plants

Because the endophytic bacteria-associated halophytic plants can help host plants tolerate salinity environments, we isolated beneficial endophytic bacteria candidates from the surface-sterilized root tissues of the salt marsh halophytic plant, *Bolboschoenus planiculmis*. We initially collected ten bacteria isolates from the *B. planiculmis* plant. Because the bacterial isolate BP-R2 exhibited high levels of tolerance to 0.5%, 5.0%, 7.0%, 9.0%, and 11.0% NaCl when grown on LB medium at 30 °C and this strain was selected for further characterization. To determine the identity of strain BP-R2, we performed fatty acid methyl esters (FAME) analysis and Biolog tests. FAME analysis revealed that strain BP-R2 had large proportions of the branched fatty acid (iso-C15:0 and anteiso-C15:0) and small amounts of strait chain fatty acids (C14:0, C16:0, and C18:0), branched fatty acids (iso-C14:0, iso-C16:0, iso-C17:0, and anteiso-C17:0), and a mono-unsaturated C16:1 ω11 cis fatty acid. The FAME analysis results of strain BP-R2 matched those of the *Bacillus* spp.

The utilization of carbonaceous compounds was further determined with Biolog tests. The substrates that could be used for growth under aerobic conditions at 30 °C were dextrin, D-maltose, D-trehalose, gentiobiose, sucrose, D-turanose, stachyose, D-raffinose, D-melibiose, γ-methyl-D-glucoside, N-acetyl-D-glucosamine, α-D-glucose, glycerol, L-alanine, L-arginine, L-aspartic acid, L-glutamic acid, L-histidine, L-pyroglutamic acid, lincomycin, D-gluconic acid, D-glucuronic acid, glucuronamide, quinic acid, L-lactic acid, citric acid, α-keto-glutaric acid, L-malic acid, γ-amino butyric acid, acetic acid, lithium chloride, potassium tellurite, and 1% and 4% NaCl. This substrate utilization pattern of strain BP-R2 showed that the similarity (SIM) index value was 0.562, which suggested its classification as *Bacillus megaterium*. Additionally, strain BP-R2 showed growth in LB medium with a pH of 5.7 and exhibited acid production with mannitol as a carbohydrate substrate, which is consistent with a previous observation of *Bacillus megaterium* [46].

To further determine the identity of strain BP-R2, we amplified and sequenced its 16S rRNA gene. The DNA sequence of the PCR product was deposited in GenBank (accession no. OP209759). The resulting sequences of the 1048-bp DNA fragments containing the partial 16S rRNA gene from strain BP-R2 showed 99.4% similarity to the same gene of *Priestia megaterium* (previously known as *Bacillus megaterium*) strain ATCC14581 [47,48], suggesting that strain BP-R2 belongs to *Priestia megaterium*.

### 3.2. Effects of Temperature, pH, NaCl Concentrations, and Carbon and Nitrogen Sources on the Synthesis of IAA by Bacteria Strain BP-R2

**Figure 1:**
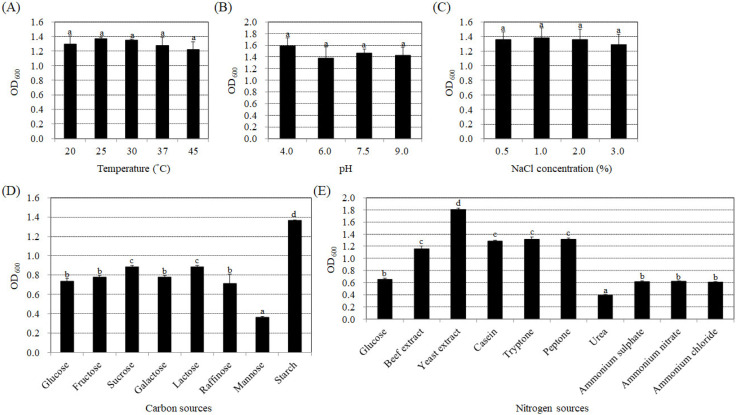
Effects of temperature, pH, NaCl concentrations, and different carbon and nitrogen sources on bacteria growth of strain BP-R2. Strain BP-R2 was cultured in Luria Broth (LB) medium (pH 7.5) at 20 °C, 25 °C, 30 °C, 37 °C, or 45 °C for 48 h to determine bacterial growth (OD_600_) (**A**). Strain BP-R2 was grown in LB medium with a pH of 4.0, 6.0, 7.5, or 9.0 at 30 °C (**B**); or in LB medium with 0.5%, 1.0%, 2.0%, or 3.0% sodium chloride (NaCl) at 30 °C (**C**) for 48 h to determine bacteria biomass (OD_600_). Strain BP-R2 was also cultivated in *Agrobacterium* minimal essential (AB-MES) medium with glucose, fructose, sucrose, galactose, lactose, raffinose, mannose, or starch at 30 °C (**D**); or in AB-MES media with 2% glucose and 0.5% beef extract, yeast extract, casein, tryptone, peptone, urea, ammonium sulphate, ammonium nitrate, or ammonium chloride at 30 °C (**E**) for 72 h to determine the bacterial biomass (OD_600_). Data are presented as the mean ± standard error (SE) from at least three independent bacterial growth experiments. Data were analyzed by Duncan tests and means with different letters were significantly different (*p* < 0.05).

**Figure 2:**
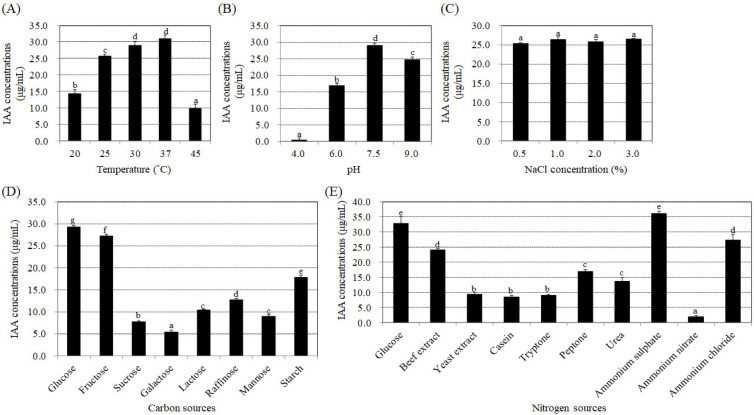
Different effects of temperature, pH, NaCl concentrations, and various carbon and nitrogen sources on indole acetic acid (IAA) production by strain BP-R2. The tested bacteria were cultured in Luria Broth (LB) medium (pH 7.5) at 20 °C, 25 °C, 30 °C, 37 °C, or 45 °C (**A**); or in LB medium with a pH of 4.0, 5.7, 6.0, 7.5, or 9.0 at 30 °C (**B**); or in LB medium with 0.5%, 1.0%, 2.0%, or 3.0% NaCl at 30 °C (**C**) for 48 h to determine IAA synthesis amounts. Strain BP-R2 was grown in *Agrobacterium* minimal essential (AB-MES) medium with 2% of various carbon sources (**D**), or in AB-MES media with 2% glucose and 0.5% of various nitrogen sources (**E**) at 30 °C for 72 h to determine IAA productions. Data are presented as the mean ± standard error (SE) from at least three independent bacterial growth experiments. Data were analyzed by Duncan tests and means with different letters were significantly different (*p* < 0.05).

To determine if the endophytic bacteria strain BP-R2 exhibits plant growth promotion ability, we examined the amount of the plant hormone, IAA, produced by strain BP-R2 under various growth conditions. Several growth parameters, such as temperature, pH, and different carbon and nitrogen sources, can influence the synthesis of IAA in bacteria [49,50]. In order to help us further understand the regulation of IAA production under different growth conditions and what kind of environmental factors that may affect IAA synthesis, we first examined the temperature effect on IAA production by strain BP-R2. The bacteria were grown in LB media (pH 7.5) at 20 °C, 25 °C, 30 °C, 37 °C, or 45 °C for 48 h. The strain grew well at temperatures ranging from 20 °C to 45 °C (Figure 1A). The bacterial cultures had the highest IAA yield of 29.0 to 31.0 μg/mL when grown at 30 and 37 °C, followed by 25 °C, and had the lowest IAA yield of 9.9 μg/mL when grown at a temperature of 45 °C (Figure 2A). We also tested the effects of pH and NaCl concentrations on IAA production by strain BP-R2. Figure 1B,C showed that strain BP-R2 grew well over a pH range of 4 to 9 and a NaCl concentration range of 0.5 to 3.0% at 30 °C for 48 h. The results of Figure 2B indicated that IAA production was the highest in the bacteria when grown at pH 7.5, followed by pH 9.0 and pH 6.0, and the lowest IAA amount of 0.4 μg/mL was from bacteria grown at pH 4.0. Figure 2C showed that IAA production was at a similar level to that of bacteria grown with the NaCl concentration range of 0.5 to 3.0% for 48 h. These data suggest that excessively high temperatures and excessively low pH may affect IAA production in strain BP-R2.

Furthermore, different carbon and nitrogen sources were used in the AB-MES medium to determine their effects on IAA production. Strain BP-R2 grew best with starch as the carbon source in the minimal medium at 30 °C for 72 h (Figure 1D), whereas the bacteria grew similarly with glucose, fructose, galactose, raffinose, sucrose, and lactose as the carbon sources (Figure 1D). The bacteria showed the least growth when cultured with mannose (Figure 1D). However, the strain produced the highest IAA amounts when grown with glucose and fructose, followed by starch and raffinose, and had the lowest IAA production when grown with galactose (Figure 2D). The results shown in Figure 1E indicated that strain BP-R2 grew best with yeast extract as the nitrogen source, followed by beef extract, casein, tryptone, and peptone. The strain showed even lower growth with ammonium sulphate, ammonium nitrate, and ammonium chloride as nitrogen sources, and grew the least with urea as the nitrogen source (Figure 1E). Surprisingly, strain BP-R2 had the highest IAA production when cultured with ammonium sulphate, followed by beef extract, ammonium chloride, urea, and peptone, and had the lowest IAA production when cultured with ammonium nitrate (Figure 2E). These data demonstrate that different carbon and nitrogen sources significantly affected IAA production when strain BP-R2 grew in the minimal medium. Furthermore, these data suggest that the bacteria growth mass might not directly correlate with IAA production levels.

### 3.3. Inoculation of Arabidopsis with Bacteria Strain BP-R2 Promoted Plant Growth under Salt and Drought Stresses

**Figure 3:**
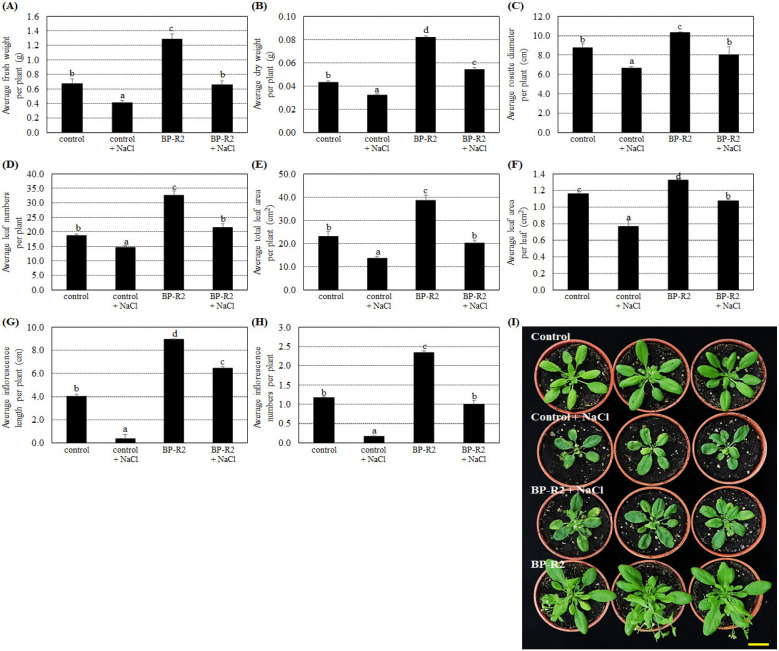
Inoculation of *Arabidopsis* with strain BP-R2 promoted its growth in comparison to the mock-inoculated control plants under salt stress treatments. Strain BP-R2 was grown in Luria Broth (LB) medium at 30 °C and then was used to inoculate *Arabidopsis* plants. After inoculation, the plants were treated with 250 mM NaCl for 5 days to create salt stress treatments and were subsequently recovered for 3 days by re-watering with distilled water. After salt stress treatments, the average values of the fresh weight per plant (**A**), dry weight per plant (**B**), rosette diameter per plant (**C**), leaf numbers per plant (**D**), total leaf area per plant (**E**), leaf area per leaf (**F**), inflorescence length per plant (**G**), and inflorescence numbers per plant (**H**) of the control and the BP-R2-inoculated plants under salt stress and nonstress conditions were recorded. Data are presented as the mean ± standard error (SE) from at least three independent bacteria inoculation experiments. More than 20 individual plants were examined for each bacteria inoculation assay. Data were analyzed by Duncan tests and means with different letters were significantly different (*p* < 0.05). (**I**) shows the top-view photographs of the mock-inoculated control and the BP-R2-inoculated plants after salt stress treatments. Yellow bar = 3 cm.

Because strain BP-R2 produced the auxin plant hormone, IAA, and tolerated relatively high concentrations of NaCl, we determined if the strain could promote plant growth under normal and high salt conditions. Wild-type *Arabidopsis thaliana* plants were inoculated with strain BP-R2 grown at 30 °C and the endophytic colonization of the plants was confirmed by re-isolating the bacteria from surface-sterilized inoculated plant tissues. The identities of the isolated bacteria were determined by sequencing and phylogenetic analysis of the 16S rRNA gene. Subsequently, various plant growth parameters were examined in *Arabidopsis* plants inoculated with strain BP-R2 and in mock-inoculated controls. After inoculation, the presence of strain BP-R2 significantly increased the average *Arabidopsis* plant fresh weight (Figure 3A), dry weight (Figure 3B), rosette diameter (Figure 3C), leaf numbers (Figure 3D), total leaf area per plant (Figure 3E), leaf area per leaf (Figure 3F), inflorescence length (Figure 3G), and inflorescence numbers (Figure 3H) as compared with mock-inoculated controls. The results shown in Figure 3I demonstrate that the leaf numbers and total leaf areas of plants inoculated with strain BP-R2 were higher and larger than the control plants, indicating that strain BP-R2 promoted *Arabidopsis* plant growth.

We further treated the control and BP-R2-inoculated *Arabidopsis* plants with 250 mM NaCl for 5 days and allowed the plants to recover for 3 days by watering with the distilled water. After salt stress treatments, the control plants showed significant reductions in the average fresh weight, dry weight, rosette diameter, leaf numbers, total leaf area per plant, leaf area per leaf, and inflorescence length (Figure 3), indicating the control plant growth parameters were affected by salt stress. The results shown in Figure 3 also demonstrated that the BP-R2-inoculated plants increased more than 1.5-fold than the control plants in different plant growth parameters, including average fresh weight, dry weight, rosette diameter, and leaf numbers when both plants were under salt stresses. These data suggest that the inoculation of *Arabidopsis* with strain BP-R2 improved plant growth under normal growth conditions and under salt stress.

**Figure 4:**
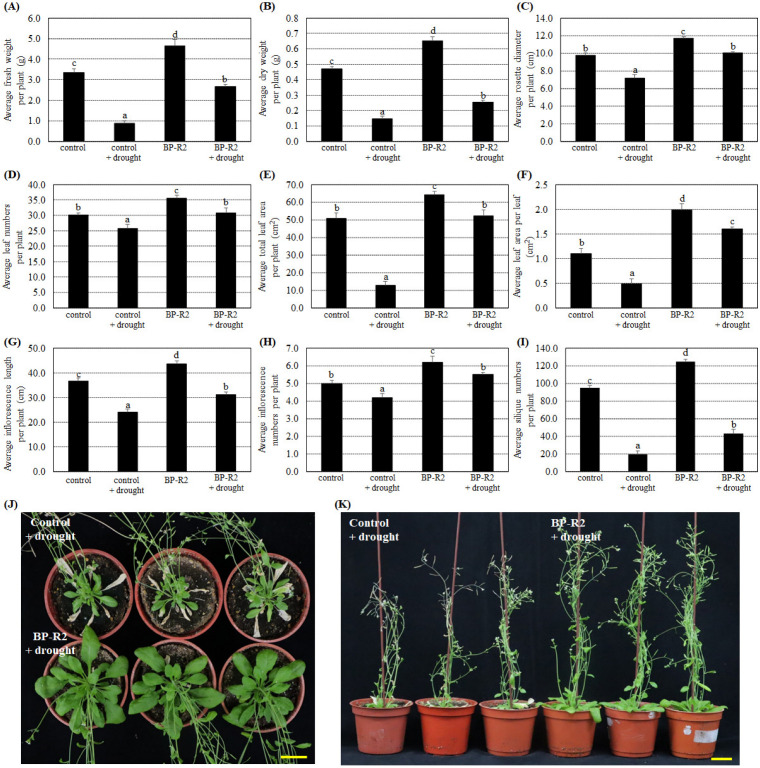
After strain BP-R2 inoculation, the *Arabidopsis* plants had better growth than the mock-inoculated control plants under drought stress treatments. Strain BP-R2 was cultured in Luria Broth (LB) medium and used to inoculate *Arabidopsis* plants. After bacteria inoculation, the plants were treated with drought stress for 10 days by withholding water and they were subsequently re-watered for 3 days for recovery. After drought stress treatments, the average values of the fresh weight per plant (**A**), dry weight per plant (**B**), rosette diameter per plant (**C**), leaf numbers per plant (**D**), total leaf area per plant (**E**), leaf area per leaf (**F**), inflorescence length per plant (**G**), inflorescence numbers per plant (**H**), and silique numbers per plant (**I**) of the control and the BP-R2-inoculated plants under drought stress and nonstress conditions were examined. Data are presented as the mean ± standard error (SE) from at least three independent bacteria inoculation experiments. More than 20 individual plants were examined for each bacteria inoculation assay. Data were analyzed by Duncan tests and means with different letters were significantly different (*p* < 0.05). (**J**) shows the top-view and Panel (**K**) shows the side-view photographs of the mock-inoculated control and the BP-R2-inoculated plants after drought stress treatments. Yellow bar = 3 cm.

Additionally, we tested whether inoculation with strain BP-R2 in plants could enhance their tolerance to other abiotic stresses. The control and BP-R2-inoculated *Arabidopsis* plants both underwent 10 days without watering to create drought stress and were recovered for 3 days with re-watering. Figure 4 results showed that the average fresh weight, dry weight, rosette diameter, leaf numbers, total leaf area per plant, leaf area per leaf, inflorescence length and numbers, and silique numbers of the control plants and the BP-R2-inoculated plants were relatively lower after treatment with drought stress, suggesting the drought stress treatments significantly inhibit plant growth. On the contrary, under drought stress, the average fresh weight, dry weight, rosette diameter, and several other plant growth parameters of BP-R2-inoculated plants were 1.2- to 3.0-fold higher than the control plants (Figure 4). These data demonstrate that inoculating *Arabidopsis* with strain BP-R2 may enhance its drought tolerance.

**Figure 5:**
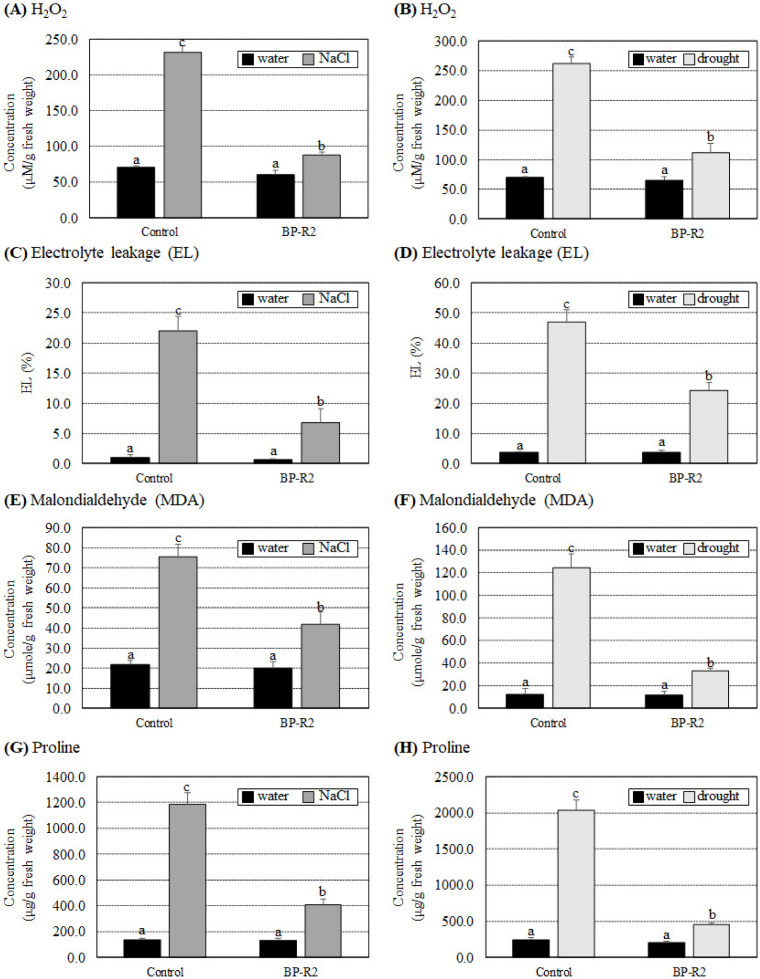
The hydrogen peroxide (H_2_O_2_), electrolyte leakage (EL), malondialdehyde (MDA), and proline concentrations were less induced in the BP-R2-inoculated *Arabidopsis* plants under salt and drought stresses. The H_2_O_2_ (**A**,**B**), EL (**C**,**D**), MDA (**E**,**F**), and proline contents (**G**,**H**) were determined in the mock-inoculated control and the BP-R2-inoculated plants under salt (**A**,**C**,**E**,**G**) and drought (**B**,**D**,**F**,**H**) stress treatments. Data are presented as the mean ± standard error (SE) from at least three independent bacteria inoculation experiments. More than 10 individual plants were examined for each bacteria inoculation assay. Data were analyzed by Duncan tests and means with different letters were significantly different (*p* < 0.05).

To further understand the possible mechanisms of growth improvement by strain BP-R2 in *Arabidopsis* plants under salt and drought stresses, we examined several biochemical parameters in the control and the BP-R2-inoculated *Arabidopsis* plants under stresses. The H_2_O_2_, EL, and MDA concentrations are major indicators of oxidative stress damage in plants under various stress conditions. The results of Figure 5A–F showed that there was no significant difference in H_2_O_2_, electrolyte leakage, and MDA concentrations in the control and the BP-R2-inoculated *Arabidopsis* plants under nonstress conditions (with water treatments). The H_2_O_2_ content increased 3.3-fold in the control plants after salt stress treatments, whereas it only increased 1.5-fold in the BP-R2-inoculated plants under the same stress treatments (Figure 5A). Similarly, under drought stress, the H_2_O_2_ concentrations showed a 3.7-fold increase in the control plants, whereas it showed only a 1.7-fold increase in the BP-R2-inoculated plants under drought stress (Figure 5B). Furthermore, the EL and MDA contents were significantly increased in the control plants under both NaCl and drought treatments, whereas the fold increases were less in the BP-R2-inoculated plants under the same stress treatments (Figure 5C–F). Plants can accumulate different metabolites, such as proline and other active molecules, to help them tolerate salt and drought stresses. The results shown in Figure 5G,H demonstrate that proline concentrations were induced more than 8.4-fold in the control plants under salt and drought stresses, while it was only induced by 2.2- to 3.0-fold in the BP-R2-inoculated plants under the same stresses. These data indicated that the BP-R2-inoculated plants showed better growth and experienced less oxidative stress damage compared to the control plants under salt and drought stresses.

### 3.4. The Endophytic Bacteria Strain BP-R2 Enhanced the Growth of Pak Choi Plants under Salt and Drought Stresses

**Figure 6:**
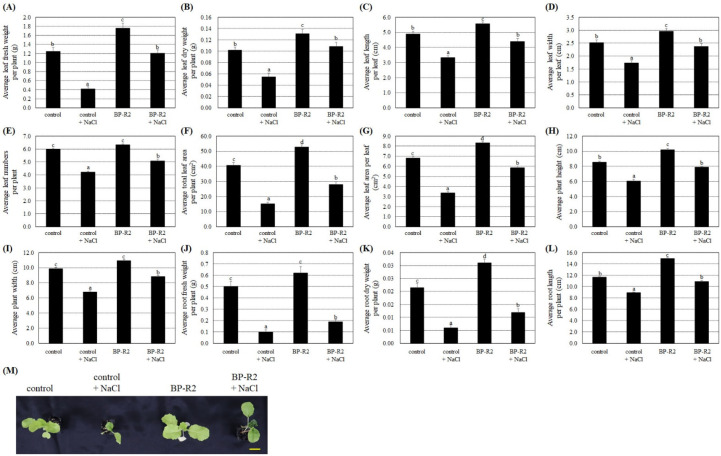
Strain BP-R2 enhanced various growth parameters of the pak choi (*Brassica rapa*) plants compared to those of the mock-inoculated control plants under salt stress. After pak choi plants were inoculated with bacteria strain BP-R2, the plants were treated with 200 mM NaCl for 5 days to create salt stress and were then recovered for 3 days by re-watering with distilled water. After salt stress treatments, the average values of the leaf fresh weight per plant (**A**) leaf dry weight per plant (**B**), leaf length per leaf (**C**), leaf width per leaf (**D**), leaf numbers per plant (**E**), total leaf area per plant (**F**), leaf area per leaf (**G**), plant height (**H**), plant width (**I**), root fresh weight per plant (**J**), root dry weight per plant (**K**), and root length per plant (**L**) of the mock-inoculated control and the BP-R2-inoculated plants under salt stress and nonstress conditions were documented. Data are presented as the mean ± standard error (SE) from at least three independent bacteria inoculation experiments. More than 20 individual plants were examined for each bacteria inoculation assay. Data were analyzed by Duncan tests and means with different letters were significantly different (*p* < 0.05). (**M**) shows the top-view photographs of the mock-inoculated control and the BP-R2-inoculated plants after salt stress treatments. Yellow bar = 3 cm.

The bacteria strain BP-R2 increased the growth of *Arabidopsis* plants under normal and stress conditions. We therefore tested its growth promotion and stress tolerance abilities on another eudicot plant from the Brassicaceae Family, namely pak choi. The four-leaf plant seedlings of pak choi (*Brassica rapa* L. R. Chinensis Group) from the *Brassica* Genus were inoculated with strain BP-R2 to examine its plant growth effects and its endophytic colonization was also confirmed by re-isolating the bacteria from inoculated plant tissues. After inoculation with strain BP-R2, the average fresh and dry weights of the aboveground leaves of the pak choi were heavier than the control plants (Figure 6A,B). Similarly, the average values of the leaf length and width, leaf numbers per plant, total leaf area per plant, and leaf area per leaf were larger in the BP-R2-inoculated pak choi in comparison to that of the control plants (Figure 6C–G). The results of Figure 6H,I showed that the average plant height and width of the BP-R2-inoculated pak choi were higher and wider than that of the control plants. Furthermore, the average root fresh weight, dry weight, and length values were higher as compared with that of the mock-inoculated control plants after the pak choi plant was inoculated with strain BP-R2 (Figure 6J–L). Figure 6 results indicated that both aerial and belowground parts of the pak choi were larger after inoculation with strain BP-R2, suggesting that the bacteria strain also promoted pak choi growth.

We next treated the pak choi seedlings with 200 mM NaCl for 5 days to establish salt stress and allowed the seedling to recover for 3 days by watering with distilled water. The results shown in Figure 6 demonstrated that, after salt stress treatment, the fresh and dry weights of both aerial and belowground parts and other plant growth parameters were relatively lower in the control plants and the BP-R2-inoculated plant in comparison to the respective plants without salt stress treatments. These data indicate that pak choi plant growths were inhibited by salt stress. Under salt stress treatment and after inoculation with strain BP-R2, the average values of the fresh and dry weights of the leaves and roots, leaf length and width, leaf numbers per plant, total leaf area per plant, and other plant growth parameters were significantly higher than those of the control plants (Figure 6). The data showed that the plant tolerance to salt stress was increased by inoculation with endophytic bacteria strain BP-R2.

**Figure 7:**
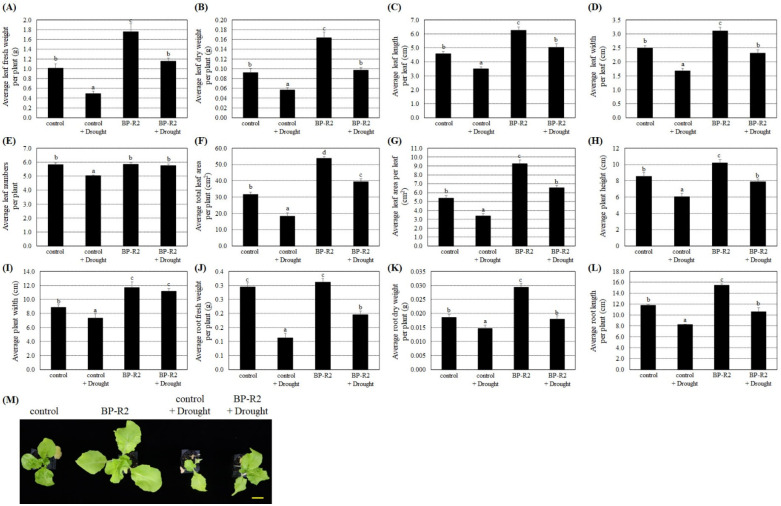
The pak choi (*Brassica rapa*) plants were larger and heavier after inoculation with strain BP-R2 under drought stress. After inoculation with strain BP-R2, plants were treated with drought stress for 5 days by withholding water and the plants were subsequently re-watered for 3 days for recovery. After drought stress treatments, the average values of the leaf fresh weight per plant (**A**), leaf dry weight per plant (**B**), leaf length per leaf (**C**), leaf width per leaf (**D**), leaf numbers per plant (**E**), total leaf area per plant (**F**), leaf area per leaf (**G**), plant height (**H**), plant width (**I**), root fresh weight per plant (**J**), root dry weight per plant (**K**), and root length per plant (**L**) of the mock-inoculated control and the BP-R2-inoculated plants under drought stress and nonstress conditions were determined. Data are presented as the mean ± standard error (SE) from at least three independent bacteria inoculation experiments. More than 20 individual plants were examined for each bacteria inoculation assay. Data were analyzed by Duncan tests and means with different letters were significantly different (*p* < 0.05). (**M**) shows the top-view photographs of the mock-inoculated control and the BP-R2-inoculated plants after drought stress treatments. Yellow bar = 3 cm.

Furthermore, the pak choi seedlings were treated with drought stress by withholding water for 5 days and then re-watering them for 3 days to recover. Under drought stresses, both the control plants and the BP-R2-inoculated plants showed lower average values of the leaf fresh and dry weights, leaf length and width per leaf, leaf numbers per plant, total leaf area per plant, leaf area per leaf, plant height and width, root fresh and dry weights, and root length as compared with those of the respective plants without drought stress treatments (Figure 7), suggesting that dehydration treatments significantly hindered plant growth. Under drought stress, the BP-R2-inoculated pak choi plants had relatively higher values of various plant growth parameters than those of the control plants (Figure 7), demonstrating that inoculation with bacteria strain BP-R2 helped the plants maintain better growth under drought stress.

**Figure 8:**
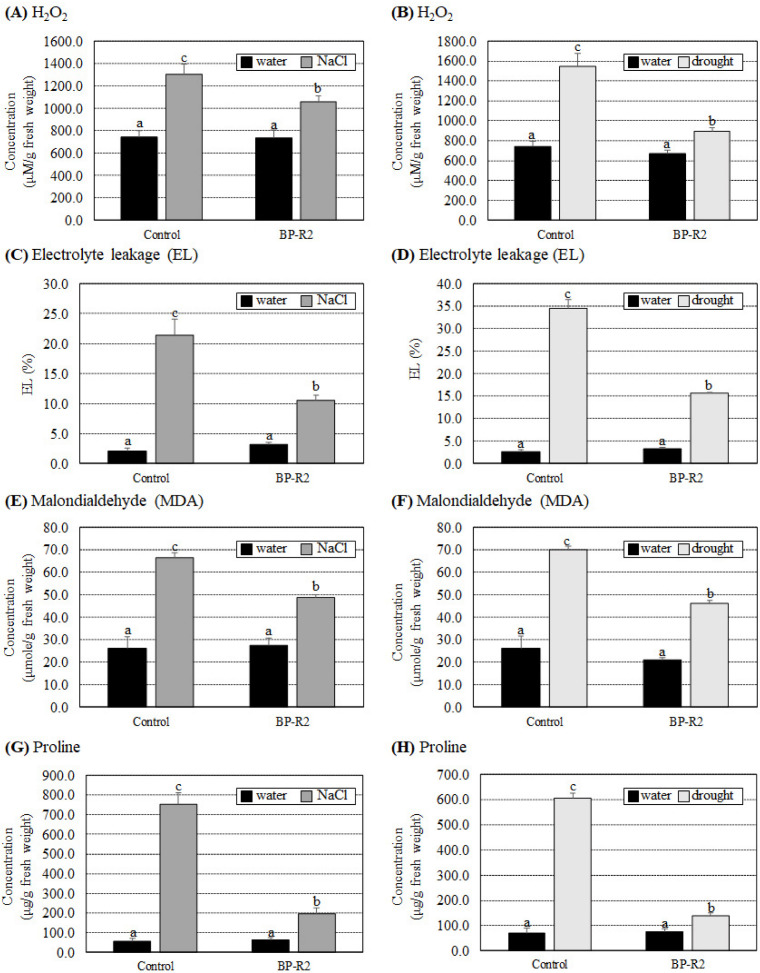
The BP-R2-inoculated pak choi (*Brassica rapa*) plants had lower induced H_2_O_2_, electrolyte leakage (EL), malondialdehyde (MDA), and proline concentrations than the mock-inoculated control plants under salt and drought stresses. The H_2_O_2_ concentrations (**A**,**B**), EL (**C**,**D**), MDA contents (**E**,**F**), and proline contents (**G**,**H**) were recorded in the mock-inoculated control and the BP-R2-inoculated plants under salt (**A**,**C**,**E**,**G**) and drought (**B**,**D**,**F**,**H**) stress treatments. Data are presented as the mean ± standard error (SE) from at least three independent bacteria inoculation experiments. More than 10 individual plants were examined for each bacteria inoculation assay. Data were analyzed by Duncan tests and means with different letters were significantly different (*p* < 0.05).

We next determined various biochemical parameters of H_2_O_2_, EL, MDA, and proline concentrations in the control and BP-R2-inoclauted pak choi seedlings after salt and drought stress treatments. The H_2_O_2_, EL, MDA, and proline contents were not significantly different between the control and the BP-R2-inoclauted pak choi plants under nonstress conditions (with water treatments) (Figure 8). Under NaCl treatments, the H_2_O_2_, EL, MDA, and proline contents increased 1.7- to 13.0-fold in the control plants (Figure 8A,C,E,G). On the contrary, in the BP-R2-inoculated pak choi plants, the H_2_O_2_, EL, MDA, and proline contents only increased by 1.4- to 3.3-fold under salt stress treatments (Figure 8A,C,E,G). When pak choi plants were treated with drought stress, increases in the H_2_O_2_, EL, MDA, and proline contents were lesser in the BP-R2-inoculated plants than in the control plants (Figure 8B,D,F,H). In summary, these results indicated that inoculation of the pak choi seedlings with strain BP-R2 enhanced plant growth and simultaneously reduced various oxidative stress damages caused by salt and drought stresses.

## 4. Discussion

Plant growth and production are substantially affected by numerous environmental factors. Salinity and drought are two major abiotic stresses encountered by plants. The application of plant endophytic bacteria to increase plant growth and abiotic stress tolerance has become a less time-consuming approach in comparison to traditional breeding and genetic engineering of abiotic stress tolerant crop varieties. Our study successfully isolated the plant endophyte *Priestia megaterium* strain BP-R2 from the salt marsh halophyte *Bolboschoenus planiculmis*. The endophytic bacteria grow in the healthy tissues of living host plants and cause no detrimental effects to the hosts. The endophytic bacteria strain BP-R2 produced significant amounts of the phytohormone auxin and may therefore greatly enhance the aerial and belowground parts of the *Arabidopsis* and pak choi plants during growth and development. Furthermore, the bacteria strain BP-R2 showed tolerances to a wide range of NaCl concentrations and helped *Arabidopsis* and pak choi plants achieve better growth under salt stress treatments. In addition, inoculation of the *Arabidopsis* and pak choi plants with strain BP-R2 assisted the plants in growing larger and better after dehydration treatments. These BP-R2-inoculated plants showed relatively lower induced H_2_O_2_, EL, MDA, and proline concentrations than the control plants under salt and drought stress treatments, which correlated well with the improved growth and development shown in these BP-R2-inoculated plants. These data demonstrated that inoculation of plants with the endophytic strain BP-R2 may alleviate the oxidative stress damage caused by salt and drought stresses.

The endophytic bacteria *Priestia megaterium* strain BP-R2 was originally known as *Bacillus megaterium*, which is a Gram-positive, mainly aerobic and spore forming bacterium that exists in widely diverse environments from plant host tissues to soil, rice paddies, dried food, seawater, honey, humans, and blood samples [48,51,52,53,54]. This bacterium was originally named because of its large size, which was almost 100 times that of *Escherichia coli* [55] and was used as a model organism for extensive studies on the sporulation process, cell biology, biochemistry, and bacteriophages of Gram-positive bacteria [51,52,53,54]. Because of its ability to produce and secrete various useful enzymes and products, including amylases, proteases, glucose dehydrogenase, penicillin amidase, vitamin B12, and a few antibiotics, *Bacillus megaterium* has been developed and industrially utilized for more than 50 years [51,53,54,56]. In a recent publication by Gupta et al. (2020) [47], extensive phylogenomic and comparative analyses were conducted with more than 300 *Bacillus*/*Bacillaceae* genomes and *Bacillus megaterium* became recognized as *Priestia megaterium* based on its multiple genomic-scale phylogenetic tree analysis results.

The *Bacillus* species can form spores, which enhance their ability to endure a broad range of stress conditions and allow their use as bacterial inoculants for agriculture and bioremediation practices [52]. Furthermore, the ability to produce the auxin, IAA, has been discovered in several *Bacillus* species, such as *Bacillus amyloliquefaciens*, *Bacillus anthracis*, *Bacillus cereus*, *Bacillus pumilus*, *Bacillus subtilis*, *Bacillus telluris*, *Bacillus thuringiensis*, and *Bacillus megaterium* [52,57,58,59,60]. Auxin is a key phytohormone in plant growth regulation, including cell enlargement, cell division, embryo development, root initiation and development, vascular tissue development, reproductive organ initiation and patterning, and phototropism [61]. The amino acid tryptophan that exists in root exudates can be absorbed and utilized by the bacteria to produce IAA via different pathways [57]. The bacteria-produced IAA can then be absorbed by plant cells and, along with the plant inherent IAA, induce various auxin signal transduction pathways leading to plant cell growth and proliferation. In this study, the endophytic bacteria strain BP-R2 synthesized up to 31.0 μg/mL IAA in the presence of tryptophan and significantly increased the biomasses of both the roots and aerial parts of the tested plant tissues. Similar to our observations, the cow dung bacterium *Bacillus megaterium* strain CDK25 can synthesize 13.8 μg/mL of IAA, increase vegetative and biological growth parameters, and enrich the mineral compositions of *Capsicum annum* L. (chili plants) [62,63]. Another endophytic *B. megaterium* strain BM18-2, isolated from the hybrid *Pennisetum* (*Pennisetum americanum* x *P. purpureum Schumach* L.), a perennial C4 bunch grass, displayed 3.0 μg/mL of IAA production, and enhanced plant growth and cadmium (Cd) absorption of hybrid *Pennisetum* plants when grown in Cd contaminated soil [64]. Additionally, the *B. megaterium* strain STB1, isolated from contaminated estuarine environment soil, contained IAA biosynthetic genes and increased the biomass of tomato plants [65]. Another endophytic bacterium, *Bacillus megaterium* strain RmBm31, isolated from the surface-sterilized root nodules of *Retama monosperma*, also had several genes involved in IAA biosynthesis pathways and significantly improved *Arabidopsis* seedling growth [66].

Our results showed that bacteria strain BP-R2 had greater IAA production at a temperature range of 25 to 37 °C and a pH range of 6 to 9. Consistent with our observations, several *Bacillus* species, including *B. siamensis*, *B. megaterium*, *B. subtilis*, and *B. cereus*, showed relatively higher IAA production at temperatures ranging from 25 to 35 °C and at a pH range of 7 to 8 [50,67]. Other bacteria isolated from the rhizosphere of *Stevia rebaudiana* showed better IAA yields at a pH range of 6 to 9 and at temperatures of 35 and 37 °C; these bacteria also improved the growth of wheat and mung bean plants [49]. Our study demonstrated that strain BP-R2 showed a difference in its preference for carbon or nitrogen sources used in IAA biosynthesis. Other studies also showed that glucose was the better carbon source for IAA production by the endophytic bacteria *B. megaterium* isolate MJHN1, the *B. cereus* isolate So3II, and the *B. subtilis* isolate Mt3b [68,69]. Several other plant growth-promoting bacteria, such as *B. megaterium*, *B. subtilis*, *B. cereus*, and *Pantoea agglomerans*, revealed their different preferences for carbon or nitrogen sources for IAA production [50,67,68,69,70,71]. These research results of endophytic bacteria strain BP-R2 may be similar to other plant growth promoting bacteria in producing and using IAA as a major factor to contribute to root growth and therefore facilitate nutrient uptake and improve aboveground tissue development.

High salinity and drought impose two major constraints to plant growth and crop yields. Excessive NaCl concentration in soils may decrease water potential and cause physiological drought conditions, which may lead to the inhibition of water and nutrient uptake in plant roots [4,5,6]. Drought stress may negatively affect cell turgor and water content, which may disturb normal plant metabolism and changes the regular morphological, physiological, and biochemical characteristics of plants [2]. Both salinity and drought stresses may induce reactive oxygen species (ROS) accumulation, such as hydrogen peroxide (H_2_O_2_), superoxide radicals, and hydroxyl radicals, which serves as a signal to trigger a defense response in plants [2,3,6]. The generation of ROS may disrupt cellular redox homeostasis, cause oxidative stress, and create severe damage by the initiation of lipid peroxidation, deterioration of membrane integrity, enhancement of electrolyte leakage, and degradation of proteins, lipids, and nucleic acids in plants [2,3,6]. In this study, the inoculation of *Arabidopsis* and pak choi plants with endophytic bacteria strain BP-R2 significantly decreased salt and drought stress-induced levels of H_2_O_2_, MDA, and electrolyte leakage in plants, which correlated well with the improved growth traits of the BP-R2-inoculated plants. In accordance with our observations, several *Bacillus* species, including *B. megaterium*, *B. thuringiensis*, *B. amyloliquefaciens*, and *B. licheniformis*, have been demonstrated to increase plant tolerance to salt or drought stress by producing IAA to increase root growth, water and nutrient uptake, compatible solute accumulation, stress-associated enzyme expression, and ROS accumulation reduction [3,7,9,10,11,13,52]. Previous studies have shown that two highly salt-tolerant *B. megaterium* strains, YC4-R4 and TG1-E1, isolated from the rhizospheric soil of a *Spartina anglica* plant in China, had abilities to promote plant growth and increase plant drought tolerance [72,73]. The genome of another *B. megaterium*, strain STB1, contained IAA biosynthetic genes and promoted tomato growth under normal and salt-stress growth environments [65]. Similar studies have demonstrated that the inoculation of *Arabidopsis* plants with the *B. megaterium* strain BOFC15 significantly increased both leaf and root biomass, primary root length, and lateral root density. Furthermore, in the BOFC15-inoculated plants, the cellular ROS levels, ion leakage, and MDA levels were all reduced under drought and polyethylene glycol (PEG)-induced stress conditions [74]. A transcriptome study of the arid condition-resistant *B. megaterium* strain FDU301 showed that several oxidative stress responsive genes were upregulated under PEG-induced arid stress conditions [75]. In summary, previous studies and our discovery suggested that the IAA synthesis and oxidative stress-reducing abilities of endophytic bacteria strain BP-R2 may collectively contribute to growth enhancement and salt and drought stress tolerances of *Arabidopsis* and pak choi plants. Furthermore, our study has discovered the potential application of the endophytic bacteria strain BP-R2 as a bioinoculant for the agricultural improvement of plant growth and stress tolerance.

## Data Availability

The data underlying this article are available in the article.
